# Factors affecting job choice among physician anesthesia providers in Uganda: a survey of income composition, discrete choice experiment, and implications for the decision to work rurally

**DOI:** 10.1186/s12960-021-00634-8

**Published:** 2021-07-28

**Authors:** Tyler J. Law, Shivani Subhedar, Fred Bulamba, Nathan N. O’Hara, Mary T. Nabukenya, Cornelius Sendagire, Adam Hewitt-Smith, Michael S. Lipnick, Janat Tumukunde

**Affiliations:** 1 Division of Global Health Equity, Department of Anesthesia & Perioperative Care, University of California San Francisco, 1001 Potrero Avenue, Building 5, Ward 3C, San Francisco, CA 94110 United States of America; 2 Institute for Global Health Sciences, University of California San Francisco, San Francisco, United States of America; 3 Department of Anesthesia and Critical Care, Faculty of Health Sciences, Busitema University, Tororo, Uganda; 4 Department of Orthopaedics, University of Maryland School of Medicine, Baltimore, MD United States of America; 5 Department of Anaesthesia, Makerere University, College of Health Sciences, Kampala, Uganda

**Keywords:** Anesthesia, Rural, Uganda, Surgery, Discrete choice experiment, Salary, Income, Incentive

## Abstract

**Background:**

One of the biggest barriers to accessing safe surgical and anesthetic care is lack of trained providers. Uganda has one of the largest deficits in anesthesia providers in the world, and though they are increasing in number, they remain concentrated in the capital city. Salary is an oft-cited barrier to rural job choice, yet the size and sources of anesthesia provider incomes are unclear, and so the potential income loss from taking a rural job is unknown. Additionally, while salary augmentation is a common policy proposal to increase rural job uptake, the relative importance of non-monetary job factors in job choice is also unknown.

**Methods:**

A survey on income sources and magnitude, and a Discrete Choice Experiment examining the relative importance of monetary and non-monetary factors in job choice, was administered to 37 and 47 physician anesthesiologists in Uganda, between May–June 2019.

**Results:**

No providers worked only at government jobs. Providers earned most of their total income from a non-government job (50% of income, 23% of working hours), but worked more hours at their government job (36% of income, and 44% of working hours). Providers felt the most important job attributes were the quality of the facility and scope of practice they could provide, and the presence of a colleague (33% and 32% overall relative importance). These were more important than salary and living conditions (14% and 12% importance).

**Conclusions:**

No providers accepted the salary from a government job alone, which was always augmented by other work. However, few providers worked only nongovernment jobs. Non-monetary incentives are powerful influencers of job preference, and may be leveraged as policy options to attract providers. Salary continues to be an important driver of job choice, and jobs with fewer income generating opportunities (e.g. private work in rural areas) are likely to need salary augmentation to attract providers.

## Introduction

There is an urgent need to address the global lack of access to safe anesthetic and surgical care, a problem faced by more than five billion individuals worldwide [1]. One of the most important contributors to this problem is the limited number of trained surgical and anesthetic providers [1]. While the recommended density of physician anesthesiologists is 4–5 per 100,000 people, many countries fall short, with the World Health Organization (WHO) African region having just 1.58 total anesthesia providers per 100,000 people [2, 3].

In Uganda, there are only 0.18 physician anesthesia providers per 100,000 people, and four of the top ten health sector vacancies are for surgery and anesthesia providers [4]. The Ugandan Ministry of Health historically aimed to address this deficit by training physician anesthetists and Anesthetic Officers (AOs, a cadre of non-physician providers with a background as a clinical officer or nurse, and with extra training in anesthesia). The government has posted vacancies for anesthesia providers throughout the country, but though newly graduating anesthesiologists have entered the workforce since 2000, the vast majority of these positions remain vacant [4, 5]. Geographic maldistribution remains an issue; nearly all of the specialist physician providers practice in the capital city of Kampala (with a few in the closest regional centers nearby), while the large majority (76%) of the population lives in rural areas [6]. At the time of the study, at least 20 physician anesthesiologists were employed at hospitals in Kampala, whereas only four were working at Regional Referral Hospitals (RRH). As each RRH is planned to have two physician anesthesiologist positions, this works out to a vacancy rate of 87.5%. Most anesthesia outside the capital is provided by AOs, yet there is high turnover, and many areas have no provider at all; the AO vacancy rate is likely > 70% [4, 5]. Although provider numbers are increasing, the urban–rural disparity persists, and the optimal strategy for addressing this is not known.

Attracting and retaining healthcare workers to rural areas is a common problem [7, 8]. Several studies have explored the determinants and effectiveness of incentives to work rurally in both high- [9–13] and low-income countries [14–19]. Clearly, salary determines the financial viability of working in a rural area (as well as the opportunity to supplement salary with an additional clinical job or nonclinical work) [20–23]. However, additional factors also strongly influence the choice of employment location, like availability of job postings, working conditions, professional support, administrative burden, opportunity for promotion, lack of career advancement opportunities, schools for children, and transportation difficulties [17, 21–27]. Neither salary nor non-monetary job factors can be ignored in designing strategies to attract workers outside urban areas.

Rurality impacts the provision of specialty care like surgery and anesthesia in specific ways. Low case volumes, availability of other members of the perioperative team, or the hospital system to support perioperative care, are unique challenges [28, 29]. Rural care faces many similar challenges in well resourced countries and in low resource countries, but differences can exist. For instance, while well resourced countries face barriers with equipment supply and low operative volumes, some low resource countries continue to struggle with inconsistent access to basic monitors, resuscitative equipment, or even electricity [23, 30, 31]. In these settings, an evidence-informed approach to human resource allocation is extremely important. Tacitly, it is suggested that government salary alone is insufficient for providers, but the actual amount of income given up when providers work only at a government job is unknown without understanding what they can make from all sources. The objectives of this study were to determine the actual composition of physician anesthesia provider incomes, the relative importance of the factors affecting recruitment and retention to rural areas, and to generate an evidence base for workforce policy.

## Methods

This study was conducted with two surveys of physician anesthesia providers in Uganda. The first survey gathered information about sources and magnitude of income from all sources, to establish reference points for salary options in the discrete choice experiment (DCE). The second survey was a discrete choice experiment to understand how important different factors were relative to each other (and to salary). This study was reviewed and approved by the University of California, San Francisco institutional review board (IRB), the Mbale Regional Referral Hospital Research and Ethics Committee, and with official support from the Association of Anesthesiologists of Uganda.

### Eligibility

Specialist physician anesthesia providers who had completed training and were currently practicing in Uganda were eligible.

### Income survey

The income survey was developed based on previous work examining income composition for healthcare workers in low-income countries [21, 22]. Categories included demographic information, government-paid and non-governmental anesthesia jobs, and non-clinical sources of income (e.g. teaching, farming, consultancy, and other personal business). Responses were reported as descriptive statistics. Work hours were asked as a measure of effort; using full or part time status, or number of days worked, was considered as a measure of effort, but there was concern that the level of effort per day or per full time job might vary considerably. Salary and work hours were asked as ranges, and the midpoint was taken for mean calculations. Respondents were also asked to report their total amount of salary earned as a single estimate. Mean salaries were weighted by number of respondents, as not all respondents held each type of job.

### Discrete choice experiment

**Fig. 1:**
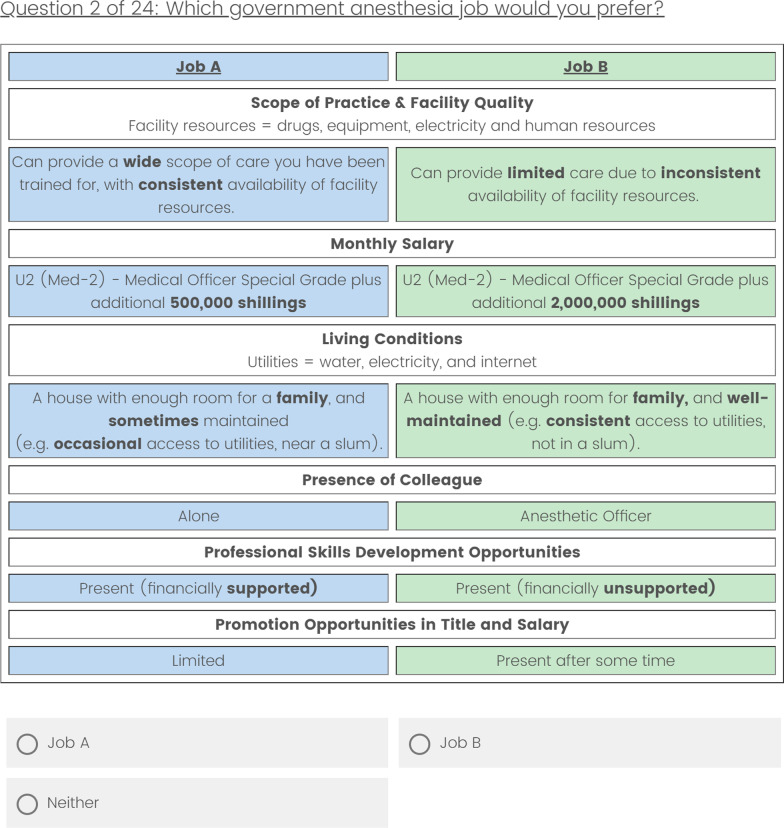
Sample Discrete Choice Experiment Question. An example of one of the 24 different job pairs. The attributes changed in each pair according to the values in Table 1

A discrete choice experiment is a type of quantitative technique used to elicit participant preferences between two or more scenarios (in this case, job choices). By presenting multiple jobs with varying attributes, the relative importance of each attribute and the trade offs required to accept a job can be determined. The DCE methodology guidelines established by WHO, the International Society for Pharmacoeconomics and Outcomes Research, and Intrahealth were followed [32–35]. Semi-structured interviews were conducted at the Association of Anesthesiologists of Uganda (AAU) national meeting to develop a list of job attributes deemed important in selecting a rural position, and participants were asked to rank attributes in order of importance. The top six attributes were selected to be included in the DCE. Based on language used in the interviews, descriptions of desirable and undesirable levels of these attributes were created (Table 1). 3,000,000 UGX was set as the upper limit of salary augmentation, representing 70–80% of a physician anesthesiologist’s government salary (3,750,000–4,200,000 UGX for Medical Officers Special Grade or Consultants at the time). This was chosen to be tangibly attractive to providers without being unrealistically high. An example of one job pair is given in Fig. 1.

**Table 1:** Discrete choice experiment attributes and levels

Attribute	Levels
**Facility quality and scope of practice** The extent to which you are able to provide the care you were trained for, given the availability of resources such as essential drugs, equipment, electricity and personnel	(a) Can provide a wide scope of care you have been trained for, with consistent availability of facility resources (e.g. drugs, equipment, electricity and human resources) (b) Can provide limited care due to inconsistent availability of facility resources (e.g. drugs, equipment, electricity and human resources)
**Salary** A change in salary with respect to the salary scale indicated above	(a) Unchanged from the current salary scale (b) Increased by 500,000 shillings (c) Increased by 1,000,000 shillings (d) Increased by 2,000,000 shillings (e) Increased by 3,000,000 shillings
**Living conditions** The type of your house in addition to access to basic utilities such as power, water, and internet	(a) A room or house for one person, poorly maintained (e.g. rare access to utilities like water, electricity, and internet, in a slum) (b) A room or house for one person, and sometimes maintained (e.g. occasional access to utilities like water, electricity, and internet, near a slum) (c) A house with enough room for a family, and sometimes maintained (e.g. occasional access to utilities like water, electricity, and internet, near a slum) (d) A house with enough room for a family, and well-maintained (e.g. consistent access to utilities like water, electricity, and internet, not in a slum)
**Presence of a colleague** Having another anesthesia provider (physician or anesthetic officer) working with you at a facility	(a) There is an anesthetic officer working at your facility (b) There is a physician anesthesiologist working at your facility (c) You are the only anesthesia provider in your facility
**Career advancement** Presence of opportunities to attend continuing medical education courses and trainings	(a) There are limited opportunities for additional training and courses (b) There are financially unsupported opportunities for additional training and courses (c) There are financially supported opportunities for additional training and courses
**Promotion opportunities** Presence of opportunities to earn a promotion with expanded responsibilities and pay after some time	(a) There is limited opportunity for promotion (b) There are opportunities for promotion after some time

### Recruitment

The AAU maintains a current list of practicing anesthesiologists in Uganda. The income survey was distributed electronically to members of the AAU mailing list and WhatsApp groups, and attendees of the annual AAU conference. Once the DCE was created, links were sent by email to the Association membership, and again via the AAU’s WhatsApp group. Reminders to encourage survey completion were sent to individual physicians by phone or WhatsApp.

Both the income survey and DCE could be collected electronically or in person, depending on participant preference. Electronic surveys contained the participant information sheet, and participants were advised that completion of the surveys constituted consent. Study personnel met the participants to complete the surveys in person if it was more convenient for them; in that case the surveys were completed either on a laptop or on paper. Participants completing the surveys in-person received the information sheet, signed a consent form, and were left alone to complete the survey. The consent forms could not be linked to the responses and were stored in a separate location from the data.

Given the sensitive nature of the salary data in particular, anonymity was important both to protect participants and ensure validity of results. Both surveys were anonymous, with no participant identifying information included (e.g. name, date of birth, name of hospital). A previously created database of the physician anesthesia providers in Uganda was used to track which physicians had reported completion of the DCE, but completion status could not be linked to responses as both the income survey and DCE were anonymized.

### Statistical analysis

There are several heuristics and guidelines to calculate a discrete choice experiment sample size [36]. One such heuristic to estimate the minimum sample size for a discrete choice experiment is to multiply 500 by the maximum number of levels in an attribute (five in our study) divided by the product of the number of choice sets (n = 24) and the number of alternatives in each choice set (n = 2). This calculation suggests that 52 respondents would provide sufficient statistical power to analyze the main effects in our study. We gain further statistical power by adding a “neither” option to each choice set.

We used the Choice Modelling platform (JMP Pro, Version 14) to develop 24 choice sets using a D-optimal, fractional factorial, balanced design to maximize design efficiency. Each choice described two hypothetical job options based on varying the levels of the six attributes. The respondents were asked to select their preferred job option for each of the 24 choice sets. All choice sets also included a “neither” option, in the event that the respondent found the jobs equally appealing or unappealing.

We used effects coding to center the levels within a given attribute around zero. The marginal utility of each attribute level was estimated using conditional logit modeling. We calculated the monetary trade-offs associated with changes in the attribute levels using marginal rates of substitution. The relative importance of each attribute was determined by the ratio of the LogWorth associated with a given attribute divided by the sum of the LogWorth values for all of the attributes. The LogWorth, also called the S-value, is the negative base-10 logarithm of the P-value for the given attribute [37]. The analyses were performed using JMP Pro Version 14 (SAS Institute, Cary, NC).

## Results

Approximately 70 providers were practicing in the country and therefore eligible to participate. A total of 37 and 47 providers participated in the income and DCE surveys for response rates of 53% and 67%. 28% and 35% of providers practiced outside Kampala. For the income survey, the mean age of the respondents was 35 years, with 35% of providers having practiced for 1–3 years (56% had practiced for 3–10 years) (Table 2). Respondent characteristics for the DCE are found in Table 2.

**Table 2:** Respondent characteristics (n, %)

	Salary	DCE
	*n* = 37	*n* = 47
Median Age (range)	32 (29–65)	33 (30–52)
Male Sex	19 (0.51)	30 (0.64)
Marital status		
Cohabitating	2 (0.06)	5 (0.11)
Divorced	1 (0.03)	–
Married	18 (0.5)	26 (0.55)
Single	15 (0.42)	16 (0.34)
Practice years		
< 1 year	–	4 (0.09)
1–3	12 (0.35)	13 (0.28)
3–5	11 (0.32)	14 (0.3)
5–10	8 (0.24)	9 (0.19)
> 10 years	3 (0.09)	7 (0.15)
Has job^a^		
Govt only	–	
Nongovt only	11 (0.30)	
Both	26 (0.70)	
Nonclinical	20 (0.54)	
Number of jobs (mean)	1.9	

^a^The clinical job categories of “government”, “nongovernment”, and “both” are exclusive; respondents could only select one. 54% also selected nonclinical work, which was in addition to their clinical jobs

Physician anesthesia providers held 1.9 jobs on average. Most reported having both government and nongovernment jobs (70%), with none reporting holding only a government job. Most providers reported earning income from nonclinical sources in addition to their clinical job (54%).

### Income composition and weekly hours worked

Physicians most commonly reported earning a monthly income of $1600–$2390 USD (UGX 6–9 million) from all sources (28%). Physicians reported earning the most income from a non-government job (50% of total income) (Table 3). On average, physicians reported working clinically an average of 30.1 h per week, with the most effort by hours in their government job (44%).

**Table 3:** Mean physician income, by job type

	Mean salary in USD (range)	%	Mean hours	%
Salary and job type				
Government job	$920 (399–1993)	0.36	35.00	0.44
Non-government job	$1020 (199–1993)	0.50	18.33	0.23
2nd NG job	$650 (199–1196)	0.05	10.00	0.06
3rd NG job	$300 (199–399)	< 0.00	NR	NR
Nonclinical work	$430 (66–1196)	0.09	10.12	0.28
Overall mean	$1900 (266–4385)	1	30.1	1

*NR* Not reported. NB: Not all respondents had all jobs, so percentages do not correspond to proportion of total means. E.g. the mean salary of physicians with nonclinical work was $430, but nonclinical work accounted for 9% of overall reported salary

### Discrete choice experiment

Of the rural job attributes presented, physicians placed the greatest relative importance on the facility quality and scope of practice, and presence of a colleague (32% and 31% relative importance), with salary and living conditions coming next (15% and 13%) (Table 4). Promotion and career advancement were less important. Respondents were given the option to choose neither job if neither were suitable; this option was much less important (3%).

**Table 4:** Relative importance of included attributes

Attribute			
Attribute	Importance (%)	95% CI	*p* value
Facility quality & scope of practice	31.9	28.8–35.0	< .01
Presence of colleague	30.9	27.9–34.0	< .01
Salary	14.6	12.3–16.9	< .01
Living conditions	13.0	10.8–15.2	< .01
Promotion	3.5	2.3–4.7	< .01
Career advancement	3.4	2.2–4.6	< .01
No job	2.7	1.6–3.7	< 0.01

Salary levels correspond to USD $0, $130, $270, $530, $800

Deterrents of job choice were quantified as the monetary compensation required for a given circumstance (Table 5). The biggest deterrent of rural jobs included in our study was being the only anesthesia provider at the hospital (UGX 3,907,827). Other major deterrents included poor and limited living conditions (UGX 2,747,004), and a limited facility and scope of practice (UGX 2,987,364).

**Table 5:** Monetary compensation associated with included attribute levels

Attribute and levels	Trade-off amount (UGX)
Facility quality & scope of practice	
Wide scope	–
Limited scope	2,987,364
Presence of colleague	
Solo	3,907,827
AO	1,184,700
MD	–
Living conditions	
Poor, limited	2,747,004
One person, inconsistent	1,913,005
Family, consistent	281,829
Family, inconsistent	
Promotion	
Limited	904,878
Available	–
Career Advancement	
Limited	1,306,194
Unsupported	411,044
Supported	–

## Discussion

This study suggests three main findings. First, salary is an important consideration in job choice. Second, non-government jobs are a significant component of salary for most providers. Third, some non-monetary incentives are as or more important motivators as salary in job selection. The majority of providers in Uganda hold both government and non-government jobs to reach their target income (around $1600-$2390 USD). In Uganda, government jobs in rural areas pay the same as the equivalent position in an urban area. The fact that no physicians held only government jobs suggests that some aspects of more rural jobs, likely salary, are insufficient. Existing literature reports that dual private–public practice is common among providers in Africa, and providers may believe or know that it will be harder to find these practice arrangements in rural areas [25, 38, 39]. A governmental “hardship” supplement for providers working in hard-to-reach areas exists, but may not overcome the reduced ability to augment income with non-government work.

The majority of providers held both government and non-government jobs, instead of working only at a higher-paying private sector non-government job. There may be a variety of reasons for this, such as insufficient availability of non-government job hours, prestige from a government position, opportunity to teach or conduct research, academic career growth, a sense of obligation, or altruism. This also suggests that providers may respond to incentives other than increased pay, a finding confirmed by the DCE. For instance, working alone was a notably important deterrent. There may be several reasons why this is, but in several other studies an important factor has been the guaranteed ability to time off, which may be difficult if there are no other providers to fill the role [17, 40, 41]. Another reason may be that the workload is unmanageable when working without much professional support [23]. Having a second provider may ameliorate both concerns.

The other important attributes in our study are in line with other studies. Facility quality has consistently been an important factor in choosing rural jobs, for instance among medical students in Uganda and clinical officers in Kenya (after tuition support, not included in our model) [17, 26]. Housing support was most important to Ethiopian doctors and Kenyan clinical officers, but somewhat less so for Ugandan medical students [18, 19]. Housing may be particularly important where infrastructure is poor [14]. There are some discrepancies with other literature, such as facility quality being less important to medical students in Laos and housing being less important to Ugandan medical students [16, 19]. This may represent how priorities change after completing training, and the realities of rural life become apparent, and may also reflect differences in factors that are important in recruitment compared to retention in rural areas (‘pull’ vs ‘stick’ factors) [23].

These nonfinancial incentives may be important levers that can be used by the government to attract providers to rural areas. For instance, working in a quality facility with a wide scope of practice and not working alone were as valuable to physician anesthesia providers as a significant salary increase. This represents a substantial policy opportunity; instead of additional funds allocated to retain a single provider, the equivalent amount may be put toward the salary of a second provider or improving the quality of the health facility. This has a dual benefit, not only providing the same retention incentive, but also improving the healthcare infrastructure at the same time, for the same cost. A practical example are nascent anesthesia ‘hubs’ developing with more than one anesthesia faculty in cities with teaching institutions, like Kabale, Mbale and Mbarara. Spent in this way, governments can obtain a significant benefit for the same healthcare spend. Additionally, while promotion and career advancement were less important than other factors, they may be more easily addressed than salary.

While it is possible for a job to attract providers without a salary increment, most other elements of the job would need to be ideal. Thus, it is unlikely that attracting and retaining providers to rural jobs without some amount of salary augmentation will be successful. Our findings are thus in line with other studies on rural retention that a package of incentives (including salary and other elements of job satisfaction) has the highest likelihood of success.

This study has some limitations. First, while the participants comprise over half of the total practicing physician anesthesia workforce, the sample size limited our ability to subsegment the population for further analysis. The design of the survey allows for the possibility of self-selection bias, but also mitigates the likely reasons for opting out (namely privacy). Second, the DCE methodology intends to capture the most important job attributes, but cannot capture all attributes important to everyone. We included attributes based on qualitative work in Uganda and prior literature, but not all important factors could be included, as each increases the required number of survey questions significantly. Attributes that were very meaningful to a subgroup (for instance, women or those with families), or several minor attributes that have collective importance would not be captured. Additionally, there may be other attributes associated with rural jobs that are unmeasured and may affect job choice. Inclusion of a rural attribute as part of the DCE would be useful in future studies. Third, though we worked with providers to define language that was meaningful in this context (including scenario examples), some individual interpretation was unavoidable. Fourth, only the aspect of rural anesthesia job demand was examined; in some places, available funding for an anesthesia job posting (rural anesthesia job supply) is also limited. Fifth, anesthetic officers, who also provide anesthesia, were not captured. In discussions with AOs, it was clear that the factors affecting their choice to work rurally were much different than physicians (e.g. much more focused on salary and workload, leading to high turnover). While their scope of practice is technically similar to physicians (providing an anesthetic for an operation), the quality of these services can be quite variable. It seemed pertinent to focus on physicians as a near term goal to also address AO quality and turnover. Nevertheless, AOs will necessarily be part of the solution to increase rural anesthesia coverage, and strategies to improve their retention and quality should simultaneously be sought..

Though this was a single-country study, the working conditions and remuneration schemes are likely to be similar to other low-income countries and other hospital based procedural specialties (e.g. surgeons and obstetricians). While the marginal utilities may differ, the finding that non-monetary incentives can be more important than salary is likely to be important throughout the region, enhancing the generalizability of these findings. Inclusion of surgeons and obstetricians in future studies will help verify if the value of incentives is consistent, and a larger sample could look at demographic subgroups. Lastly, studies on the total cost of incentive packages should be performed to enable informed policymaking for individual countries.

## Data Availability

Data from this study is not available for sharing, out of prior agreement with the Association of Anesthesiologists of Uganda, due to the sensitive nature of the data and small data set.

## Appendix

See Table

**Table. 6:** Marginal utility of included attributes and levels

Attribute	Level	Marginal utility	95% CI	*p* value
Levels				
Facil levels ity & scope	Wide scope	0.51	0.41 to 0.61	< 0.01
	Limited scope	− 0.51	− 0.61 to − 0.41	
Salary^+^	Per 1,000,000 UGX	0.34	0.24 to 0.43	< 0.01
Living conditions	Poor, limited	− 0.51	− 0.70 to − 0.33	< .01
	One person, inconsistent	− 0.23	− 0.41 to − 0.05	
	Family, inconsistent	0.42	0.25 to 0.59	
	Family, consistent	0.32	0.13 to 0.51	
Colleague	AO	0.17	0.01 to 0.32	< 0.01
	MD	0.58	0.44 to 0.73	
	Solo	− 0.75	− 0.91 to − 0.59	
Career advancement	Limited	− 0.25	0.40 to − 0.10	< 0.01
	Unsupported	0.05	− 0.10 to 0.19	
	Supported	0.20	0.06 to 0.34	
Promotion	Limited	− 0.16	− 0.25 to − 0.06	< 0.01
	Available	0.16	0.06 to 0.25	

^6^.

## Abbreviations

DCE: Discrete Choice Experiment
USD: US Dollars
UGX: Uganda Shillings
WHO: World Health Organisation
